# Evolutionary trend analysis of the pharmaceutical management research field from the perspective of mapping the knowledge domain

**DOI:** 10.3389/frhs.2024.1384364

**Published:** 2024-07-11

**Authors:** Junkai Shen, Sen Wei, Jieyu Guo, Shuangshuang Xu, Meixia Li, Dejiao Wang, Ling Liu

**Affiliations:** ^1^ School of Pharmacy, Henan University, Kaifeng, China; ^2^ Department of Pharmacy, Zhengzhou Shuqing Medical College, Zhengzhou, China

**Keywords:** pharmaceutical management, mapping knowledge domain, co-occurrence analysis, co-citation analysis, burst detection

## Abstract

**Background:**

Pharmaceutical management is a new frontier subject between pharmacy, law and management, and related research involves the whole process of drug development, production, circulation and use. With the development of medical systems and the diversification of patients’ drug needs, research in the field of pharmaceutical management is becoming increasingly abundant. To clarify the development status of this field, this study conducted a bibliometric analysis of relevant literature in the field based on the knowledge graph method for the first time and explored the evolutionary trends of research hotspots and frontiers.

**Methods:**

Literature was obtained from the Web of Science Core Collection database. CiteSpace 6.2.R4 (Advanced), VOSViewer, Scimago Graphica, Pajek and the R programming language were used to visualize the data.

**Results:**

A total of 12,771 publications were included in the study. The publications in the field of pharmaceutical management show an overall increasing trend. In terms of discipline evolution, early research topics tended to involve the positioning of pharmacists and pharmaceutical care and the establishment of a management system. From 2000 to 2005, this period tended to focus on clinical pharmacy and institutional norms. With the development of globalization and the market economy, research from 2005 to 2010 began to trend to the fields of drug markets and economics. From 2010 to 2015, research was gradually integrated into health systems and medical services. With the development of information technology, after 2015, research in the field of pharmaceutical management also began to develop in the direction of digitalization and intelligence. In light of the global pandemic of COVID-19, research topics such as drug supply management, pharmaceutical care and telemedicine services under major public health events have shown increased interest since 2020.

**Conclusion:**

Based on the knowledge mapping approach, this study provides a knowledge landscape in the field of pharmaceutical management research. The results showed that the reform of pharmacy education, the challenge of drug management under the COVID-19 pandemic, digital transformation and the rise of telemedicine services were the hot topics in this field. In addition, the research frontier also shows the broad prospects of the integration of information technology and pharmaceutical management, the practical value of precision pharmaceutical services, the urgent need of global drug governance, and the ethical and legal issues involved in the application of artificial intelligence technology in drug design, which points out the direction for the future development of pharmaceutical practice.

## 1 Introduction

In the era of information explosion, the accumulation and dissemination of scientific knowledge has accelerated unprecedentedly. In the face of massive literature, how to effectively mine, understand and visualize the knowledge structure has become an important topic of academic research, and scientific knowledge mapping technology has become an important means to solve this problem (1). Research on scientific knowledge mapping originated in the field of information visualization, which mainly reveals the structure, evolution and cross-fertilization process of scientific knowledge by mining the intrinsic connections and dynamic evolution of literature data (2). With the rapid development of big data and artificial intelligence technology, the application scope of these methods are constantly expanding (3). In particular, the emergence of tools such as CiteSpace (2), VOSviewer (2), Pajek (4), and R-tool (5) has led to the widespread application of scientific knowledge mapping technologies in many disciplines, which provides new perspectives for researchers to understand in depth the development of disciplines and cutting-edge dynamics.

As a new interdisciplinary discipline, pharmaceutical management aims to ensure the safety, effectiveness, economy and rationality of public medication through the supervision of the whole life cycle of drugs. With the development of the pharmaceutical industry and changes in the policy environment, research in the field of pharmaceutical management has increased, and research hotspots and trends are constantly changing. Therefore, sorting out the evolutionary trends of research topics in the field of pharmaceutical management from the massive literature is highly important, as is clarifying the current research hotspots and frontiers, which are important for promoting the theoretical and practical development of the field. At present, scholars have used the scientific knowledge mapping method to explore the development trends of “pharmacoeconomics” (6) and “pharmaceutical care” (7), but no research has been conducted on trends in the field of pharmaceutical management. Therefore, to fill this gap, the purpose of this study is to analyze the relevant literature in the field of pharmaceutical management through the use of the scientific knowledge mapping method to identify research trends in this area and to solve the following problems:

Q1: What is the global development trend in the field of pharmaceutical management based on the information from published literature?
Q2: What are the most prolific and influential countries/regions and institutions in this field?
Q3: What are the main research directions and hotspots? How did they change over time?
Q4: What are the current research frontiers in the field? What are the potential hotspots for the future?

## 2 Methods

### 2.1 Data sources and retrieval strategies

The method used for data analysis is closely related to the data composition. In addition to classifying and indexing an academic paper according to its own database, indexing databases usually include all the relevant literature except for the text. Considering the integrity of the data structure and the level of the paper, this study selected the Web of Science Core Collection (WoSCC) as the data source.

The retrieval criteria were as follows: (TS = “Drug Management” OR “Pharma* Administration” OR “Pharma* Management” OR “Medication Administration” OR “Pharma* Care” OR “Pharma* Service” OR “Drug Regulation” OR “Pharmacy Regulation” OR “Medication Regulation” OR “Pharma* Policy” OR “Drug Policy” OR “Pharmaceutical legislation”). To capture as many data sources as possible, the wildcard character (*) that could be substituted for any other characters and allows variable endings of keywords was used.

### 2.2 Data collection and analysis

**Figure 1:**
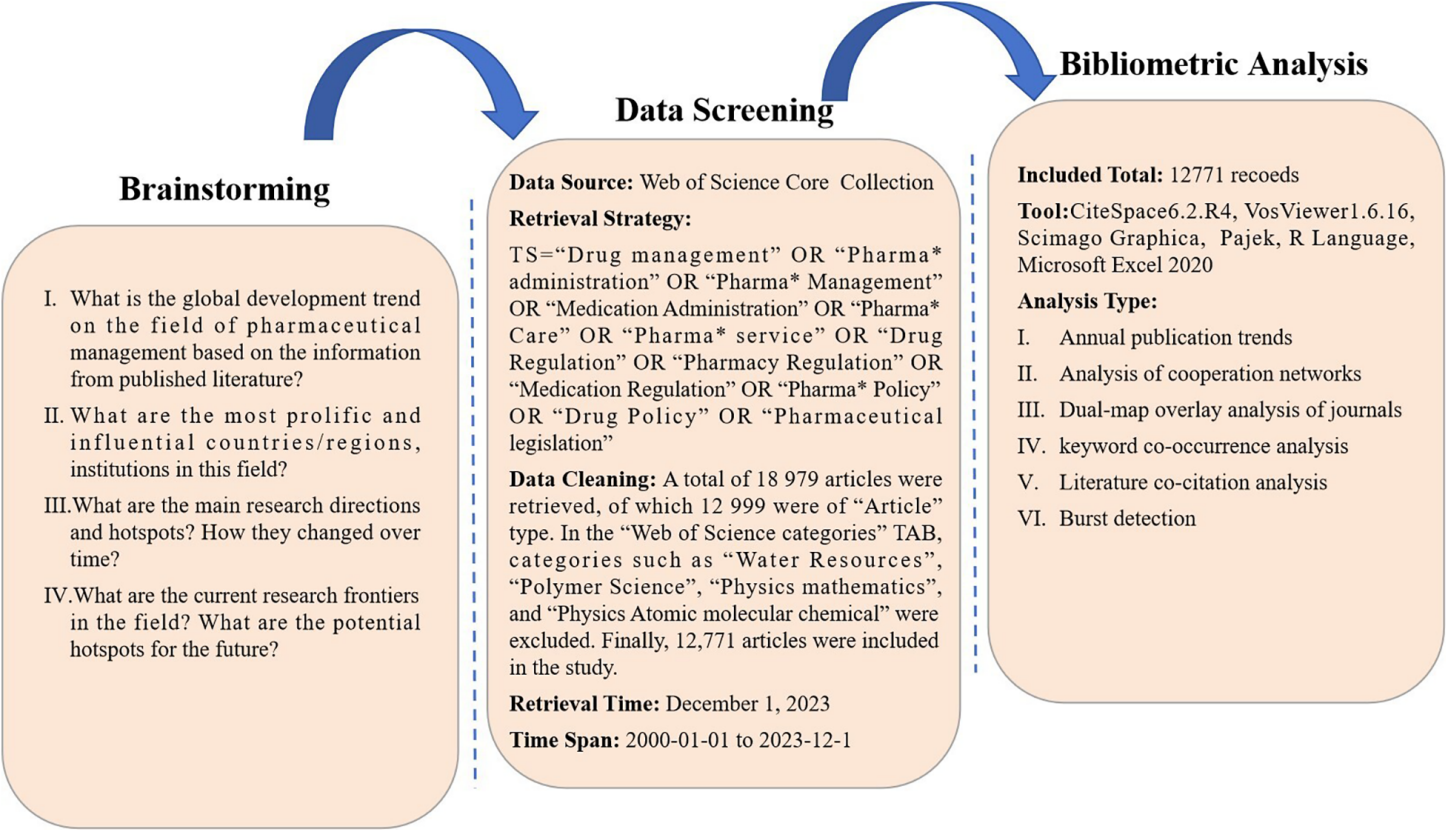
Conceptual design and flowchart of the study.

All the literature data for this study were downloaded independently from the Web of Science Core Collection database on December 1, 2023, by the author (JK Shen). The process of cleaning the literature data is shown in Figure 1. The bibliometric analysis in this paper included academic community analysis, dual-map overlay analysis of journals, keyword co-occurrence analysis, literature co-citation analysis and burst detection (8).

Visualization of the academic community analysis by VOSviewer and Scimago Graphica; visualization of dual-map overlay analysis of journals by VOSviewer and Pajek; and visualization of keyword co-occurrence analysis by VOSviewer, Pajek and the R programming language. Literature co-citation analysis and burst detection were visualized by CiteSpace 6.2.R4 (Advanced).

## 3 Results

### 3.1 Annual publication trends and dual-map overlay analysis

**Figure 2:**
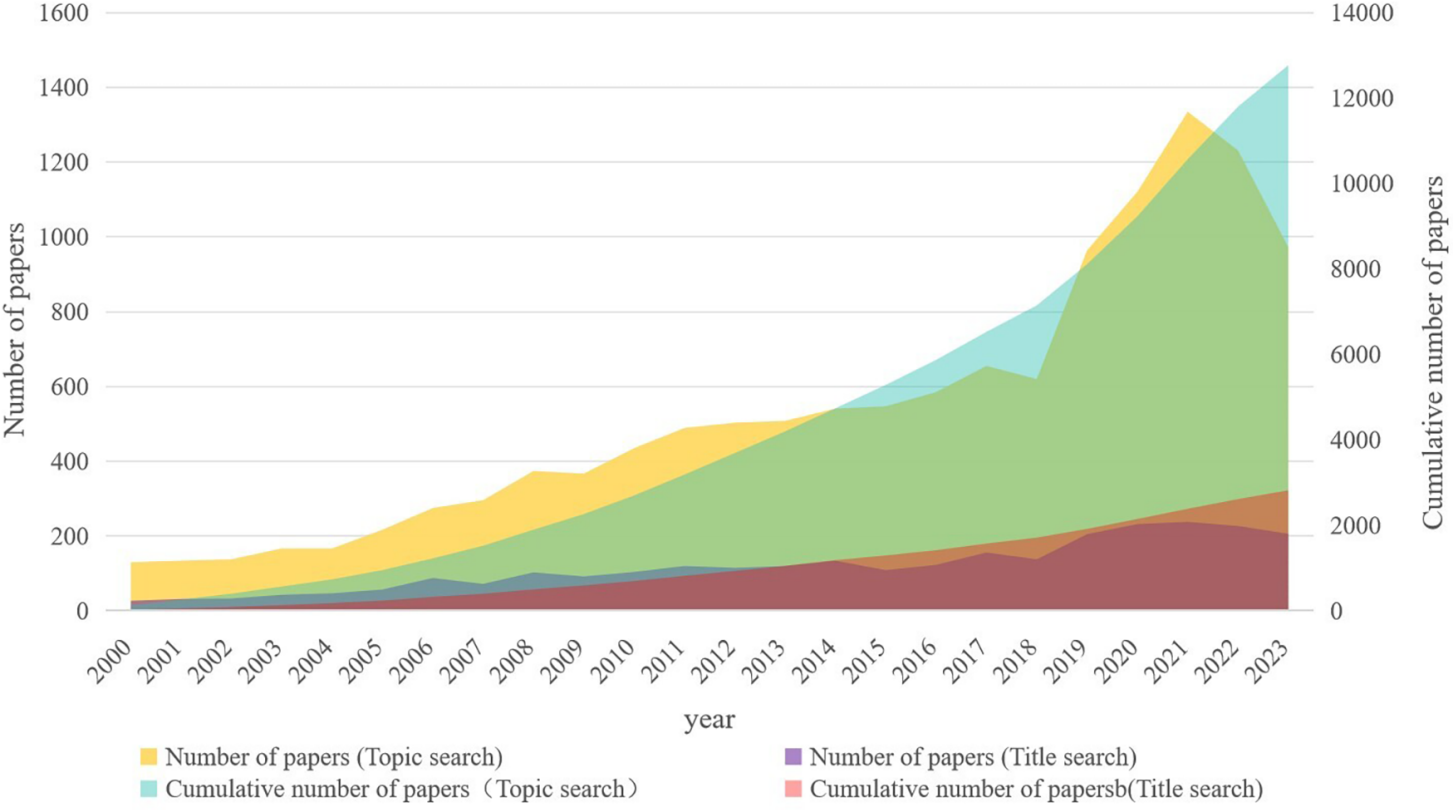
Publication trends of papers in WoSCC database. The publication of papers first started in 2000 and reached a peak of 1,335 in 2021.

The number of annual publications in the field of pharmacy administration in the WoSCC database is shown in Figure 2. The time span of the literature was from 2000 to 2023. Overall, publications in this field showed a steady growth trend, with a peak of 1,335 articles published in 2021 and a total of 12,771 articles published in the past 24 years.

**Figure 3:**
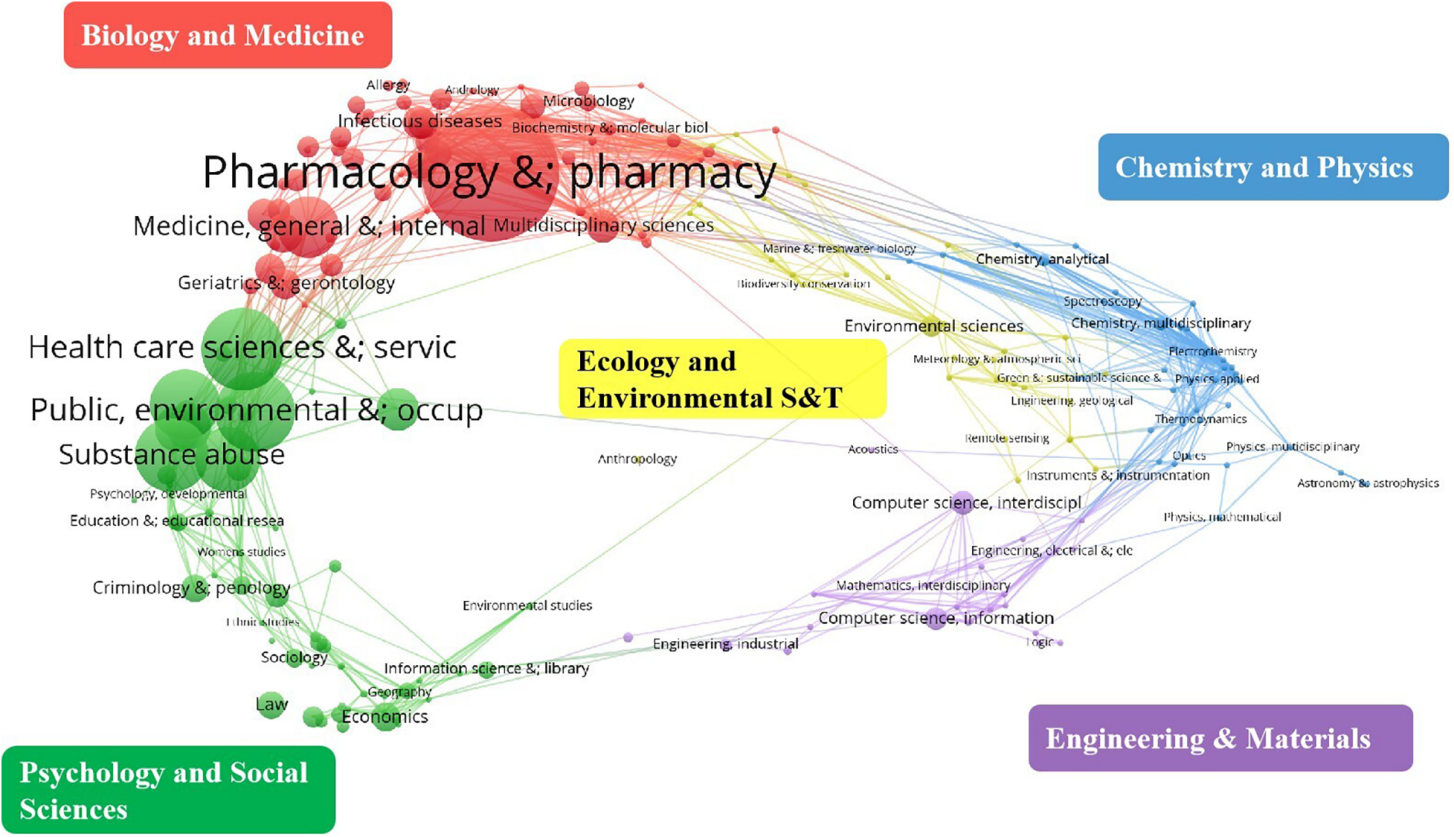
Dual-map overlay analysis of journals of pharmaceutical management fields. The literatures are mainly distributed in two subject clusters of “Biology and Medicine” and “Psychology and Social Sciences”.

Based on the Web of Science subject classification standard, all the literature was analyzed via dual-map overlay analysis. The visualization of the result was achieved through VOSviewer and Pajek. The results revealed 12,771 papers distributed across 175 different fields, as shown in Figure 3. The literature in this field is primarily distributed across two major disciplinary groups: Biology and Medicine, Psychology and Social Sciences. Among the subfields, the most widely distributed areas include Pharmacology & Pharmacy, Health Care Sciences Services, Public Environmental Occupational Health, Substance Abuse, Nursing, Health Policy Services, Medicine General Internal, Psychiatry, Education Scientific Disciplines and Medical Informatics.

### 3.2 Analysis of national and institutional cooperation

There are many forms of scientific research cooperation, and the scientific research cooperation mentioned in this article refers to the countries or scientific research units that appear at the same time in an article; thus, we determined that there is a cooperative relationship between them (9). The analysis helps us understand the core research groups and cooperation in this field, which can provide a reference for the introduction of academic resources or subject cooperation in the future. We visualized this cooperative relationship through the Scimago Graphica tool.

**Figure 4:**
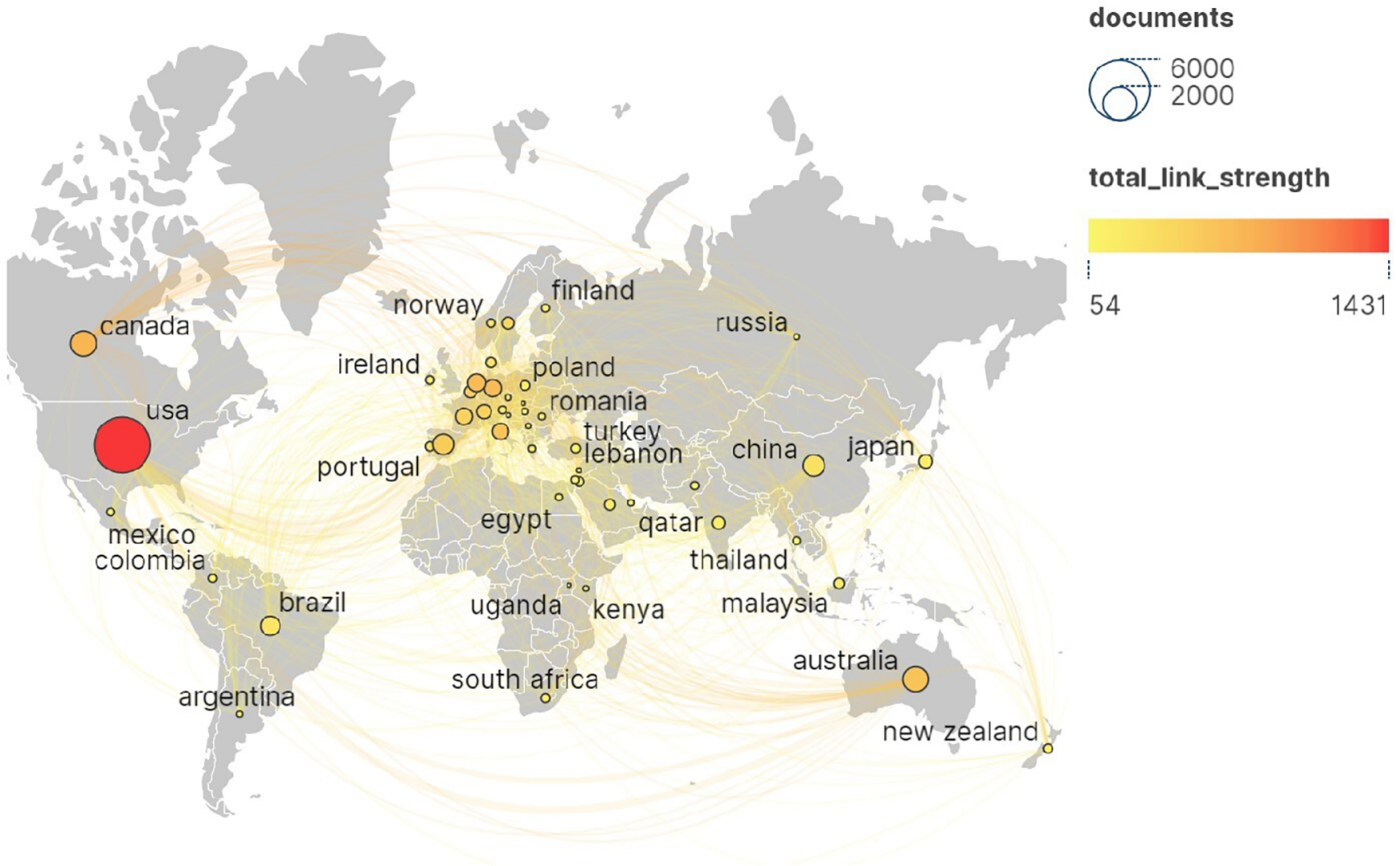
National collaborative network for papers in the field of pharmaceutical management. The United States ranked first in the world in terms of the number of publications (*n* = 4,557, 35.68%) and citations (*n* = 106,685). The size of the nodes in the figure represents the number of publications, the color represents the total link strength, and the lines between nodes represent the cooperative relationships. “Total link strength” is a key indicator used to quantify the total strength of the connection between specific nodes in the network and other nodes, with greater value indicating closer cooperation.

Figure 4 shows the national cooperation network of papers in this field. The size of the nodes in the figure represents the number of publications, the color represents the total link strength, and the lines between nodes represent the cooperative relationships. “Total link strength” is a key indicator used to quantify the total strength of the connection between specific nodes in the network, such as authors, countries, institutions, etc., and other nodes. The larger the value, the closer the cooperation relationship. According to the results, these studies involved 166 countries or regions, among which the United States ranked first in the world in terms of the number of publications (*n* = 4,557, 35.68%) and citations (*n* = 106,685), and formed a radiation to other countries and regions, which had an important academic influence in the field of pharmaceutical management research. The other countries with more than 300 publications were England (1,343, 10.52%), Canada (943, 7.38%), Australia (908, 7.11%), China (671, 5.25%), Spain (623, 4.88%), Brazil (545, 4.27%), the Netherlands (480, 3.76%), Germany (436, 3.41%), France (409, 3.20%) and Italy (375, 2.94%). Overall, research in the field of pharmaceutical management has formed a close and extensive cooperation network around the world.

**Figure 5:**
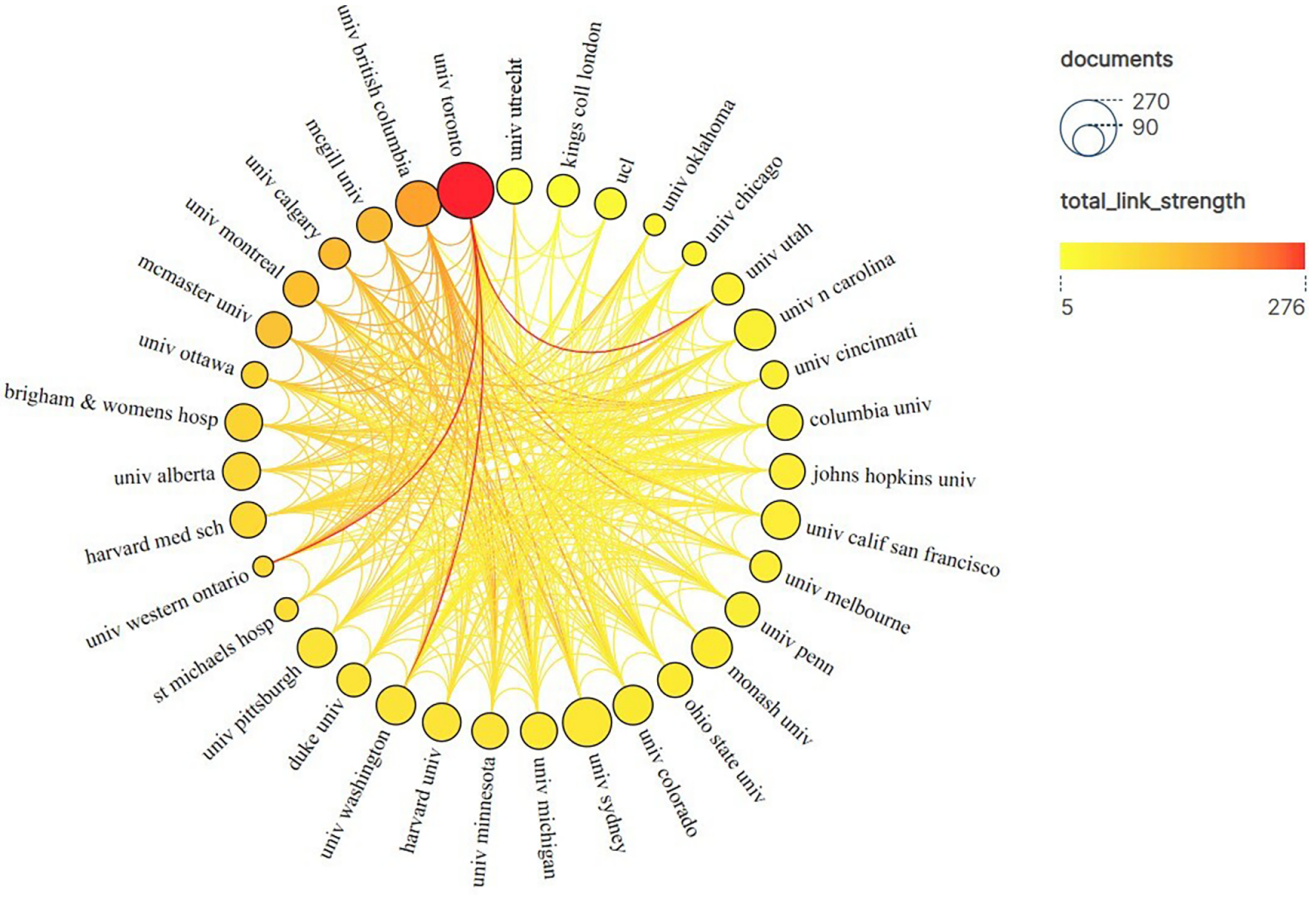
Institutional collaboration network for papers in the field of pharmaceutical management. Univ Toronto ranked first in terms of the number of publications (*n* = 242) and citations (*c* = 8,973) among all the institutions. The size of the nodes in the figure represents the number of publications, the color represents the total link strength, and the lines between nodes represent the cooperative relationships. “Total link strength” is a key indicator used to quantify the total strength of the connection between specific nodes in the network and other nodes, with greater value indicating closer cooperation.

Figure 5 shows the network of institutional collaborations for papers in this field. According to the results, Univ Toronto ranked first in terms of the number of publications (*n* = 242) and citations (*c* = 8,973) among all the institutions, indicating its high academic influence in this field. The following institutions are included: the University of Sydney (*n* = 193, *c* = 3,253), the University of British Columbia (*n* = 158, *c* = 5,133), the University of North Carolina (*n* = 128, *c* = 2,360), Monash University (*n* = 126, *c* = 2,037), the University of Colorado (*n* = 120, *c* = 3,480), the University of Washington (*n* = 118, *c* = 4,144), the University of Pittsburgh (*n* = 118, *c* = 2,608) and the University of California San Francisco (*n* = 115, *c* = 4,254), most of which can be found in the United States.

### 3.3 Keyword co-occurrence analysis

**Figure 6:**
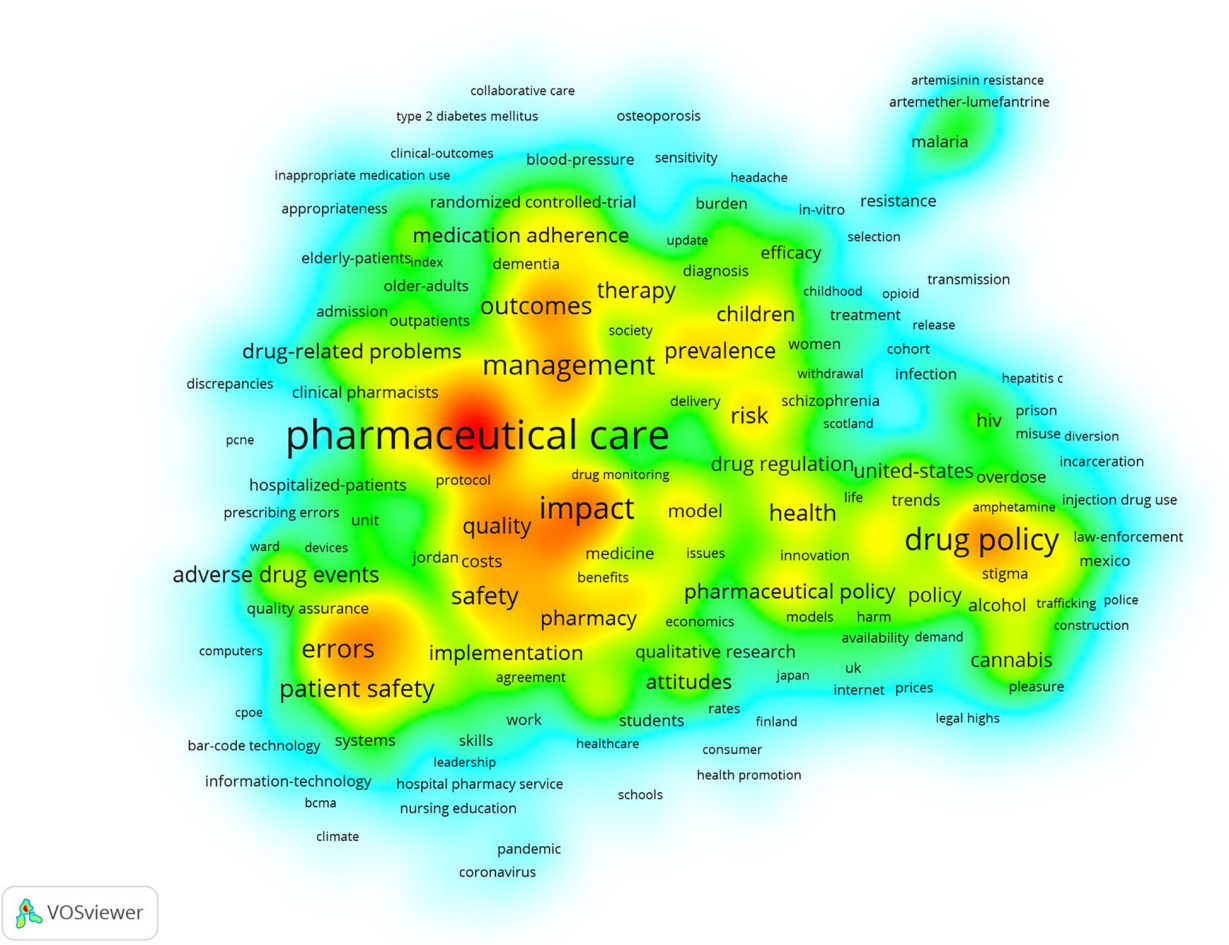
Keyword frequency density distribution of papers in the field of pharmaceutical management. Keywords that tend to occur in the middle of yellow areas have a higher co-occurrence frequency, while the green areas at the edge have the opposite trend. Keywords such as “pharmaceutical care”, “drug policy”, “medication errors”, “patient safety”, “adherence”, “pharmacy”, “interventions”, “outcomes”, “costs”, “prevalence” and “adverse drug events” had high co-occurrence frequencies.

**Figure 7:**
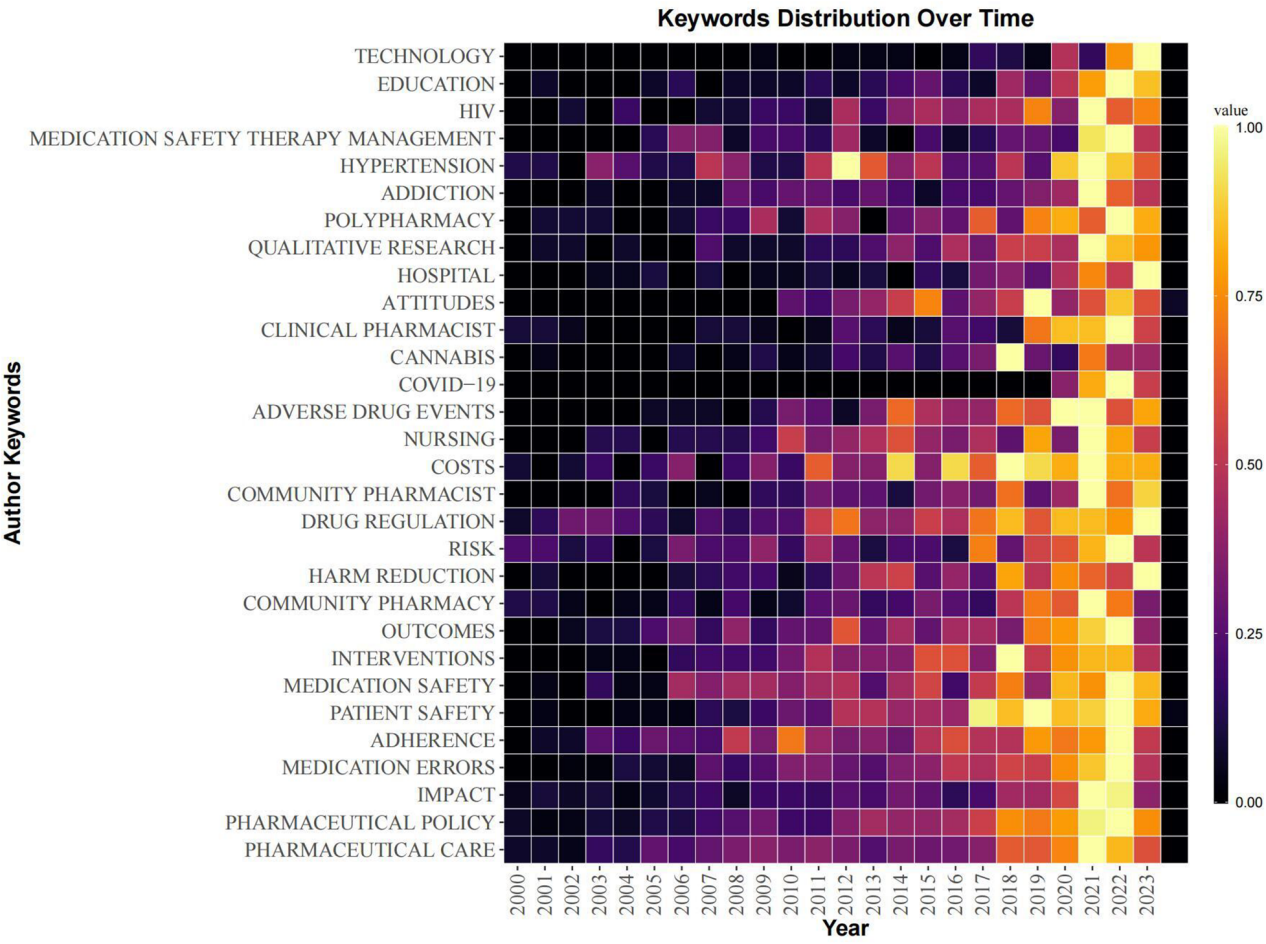
Author keywords heat map of papers in the field of pharmaceutical management. The figure shows the trend of the popularity of author keywords over time, and the color of the square represents the frequency of author keywords. The larger the value, the greater the attention of the keyword at this stage.

Keywords are often regarded as highly condensed versions of a paper and can reflect the main object and core issues of a scholar's research. Through keyword co-occurrence analysis, we identified the core keywords in this field and their positions in the network and subsequently revealed the current research hotspots (10). The results were visualized by VOSviewer (Figure 6) and R-bibliometrix (Figure 7).

Figure 6 shows the distribution of the keyword frequency density of papers in the field of pharmaceutical management. In the figure, keywords that tend to occur in the middle of yellow areas have a higher co-occurrence frequency, while the green areas at the edge have the opposite trend. The results showed that keywords such as “pharmaceutical care”, “drug policy”, “medication errors”, “patient safety”, “adherence”, “pharmacy”, “interventions”, “outcomes”, “costs”, “prevalence” and “adverse drug events” had high co-occurrence frequencies.

Although this study hopes to describe the distribution of hot topics in the field of pharmaceutical administration based on the distribution of keyword word frequency, there are still some limitations in the processing of keyword co-occurrence analysis. Specifically, because the keywords in the literature are subjectively marked by the authors, there are more synonyms, such as “drug policy” and “drug management policy”. In view of the large number of keyword nodes, manual merging and standardization of them one by one is not only very cumbersome in operation, but also may introduce new subjective judgment errors. Therefore, this study did not merge all synonyms, but retained the diversity of the original data. This approach may result in a slightly fragmented representation of some research topics in the co-occurrence network and may slightly underestimate the actual attention paid to some core topics. Although the preliminary analysis shows that these subtle keyword differences have limited impact on the overall research hotspot identification and will not fundamentally change the main findings of the study, it is still necessary to consider the use of more advanced natural language processing technology to optimize the keyword processing process in future studies to improve the accuracy and consistency of the analysis.

In addition, in order to reflect the changing trend of keyword popularity over time, we plotted author keyword heat map based on R-bibliometrix to detect the hot research topics in recent years. Because the information provided by the keywords of the literature is relatively limited, the results on research hotspots provided by a single analysis may also be biased, so this analysis can also be mutually corroborated and supplemented with the results of subsequent literature co-citation analysis. As shown in Figure 7, the keywords “education”, “HIV”, “medication safety therapy management”, “clinical pharmacist”, “cannabis” and “COVID-19” have shown high heat in recent years.

Since the information provided by the keywords is relatively limited, the literature under the keyword nodes is used to reflect the specific content of the keywords. In addition, the number of literature under some nodes is huge, so we filtered the strongly relevant literature, latest literature and highly cited literature as the representative documents under the author keyword nodes through the “sort” tab of Web of Science. The author keyword node information is shown in Table 1. There were 147 papers under the “education” node, among which the most relevant literature was on clinical pharmacist education in China (11). The latest literature is an investigation of the effect of implementing instructional games on the learning outcomes of a pharmacy practice course (12). The highly cited literature involved a study on the positive impact of pharmacists in the transitional care process of high-risk patients (13). Under the “HIV” node, the most relevant literature reported Brazil's efforts and contributions in changing the global specification of essential medicines and its experience and lessons in the treatment of AIDS (14). Recent literature under this node reports the potential positive impact of drug consumption rooms on reducing high risk behaviors of drug users for human immunodeficiency virus (HIV) and hepatitis C (15). The highly cited literature is a clinical study of adherence to highly active antiretroviral therapy (HAART) in children (16).

**Table 1:** A breakdown of the literature information under hot keyword nodes.

Keyword	Counts	Representative literature^a^	
Education	147	Strongly correlated citation	Ryan et al. (11)
		Latest citation	Dabbous et al. (12)
		Highly cited citation	Phatak et al. (13)
HIV	258	Strongly correlated citation	Nunn et al. (14)
		Latest citation	Lalanne et al. (15)
		Highly cited citation	Van Dyke et al. (16)
Medication safety therapy management	277	Strongly correlated citation	Jackson et al. (17)
		Latest citation	Zavaleta-Monestel et al. (18)
		Highly cited citation	Allemann et al. (19)
Clinical pharmacist	308	Strongly correlated citation	Verrue et al. (20)
		Latest citation	Cheng et al. (21)
		Highly cited citation	Spinewine et al. (22)
Cannabis	291	Strongly correlated citation	Jochen et al. (23)
		Latest citation	Wilson et al. (24)
		Highly cited citation	Smart et al. (25)
COVID-19	239	Strongly correlated citation	Poppe et al. (26)
		Latest citation	Zhang et al. (27)
		Highly cited citation	Cadogan et al. (28)

^a^ The representative literatures were filtered by the “sort” function in Web of Science.

In the node “medication safety therapy management”, the most relevant literature designed questionnaires from the preferred terms related to pharmaceutical care, service nature, perceived benefits and barriers and discussed the construction of the pharmaceutical care framework (17). The most highly cited publication was the definition of PCNE for pharmaceutical care (19). Recent literature provides insight into the practices of clinical pharmacists in pharmaceutical care in the Latin American region (18). In the “Clinical Pharmacist” node, the most relevant literature was a study on drug management in nursing homes, highlighting the contribution of clinical pharmacists in reducing the medication error rate (20). The latest literature introduces clinical pharmacists in central nervous system infection (CNSI) for the treatment of infections in patients with positive effects (21). The highly cited literature was a randomized controlled trial that explored the impact of a collaborative approach on prescription quality in geriatric inpatients (22).

In the “cannabis” node, the most relevant literature involved the study of policy making for medical cannabis management in schools (23). The latest literature explores the legalization of prescription use of medical cannabis in the UK (24). The highly cited literature explores the question of how cannabis policy in the United States affects substance use and provides early evidence on the effects of cannabis legalization on cannabis use, cannabis use disorders, and other substance use (25). In the “COVID-19” node, the most relevant literature explored the impact of nondrug policy interventions on COVID-19 transmission in three Colombian cities (26). The latest literature is a cross-sectional survey of patients with fever on their knowledge, attitudes and behaviors toward over-the-counter antipyretics (OTC) (27). The highly cited literature discussed the role and activities of community pharmacists in the public health crisis, with special emphasis on their contribution to the prevention of COVID-19 (28).

### 3.4 Literature co-citation analysis

Co-citation analysis is a quantitative method in which cocitation information is used for in-depth mining to reveal the knowledge structure and internal relationships of a subject domain. Because the knowledge base of a certain field is precisely based on the cocited literature, some cited studies with high citation and high mediation centrality also constitute the core knowledge base of the field. The mapping of cited literature to cited literature also reflects the mapping of the knowledge base to the research frontier. In addition, the cocitation relationship of literature often involves a dynamic process, so co-citation analysis can capture the development and evolution of this field (29). The visualization of the result is based on CiteSpace 6.2.R4 (Advanced).

**Figure 8:**
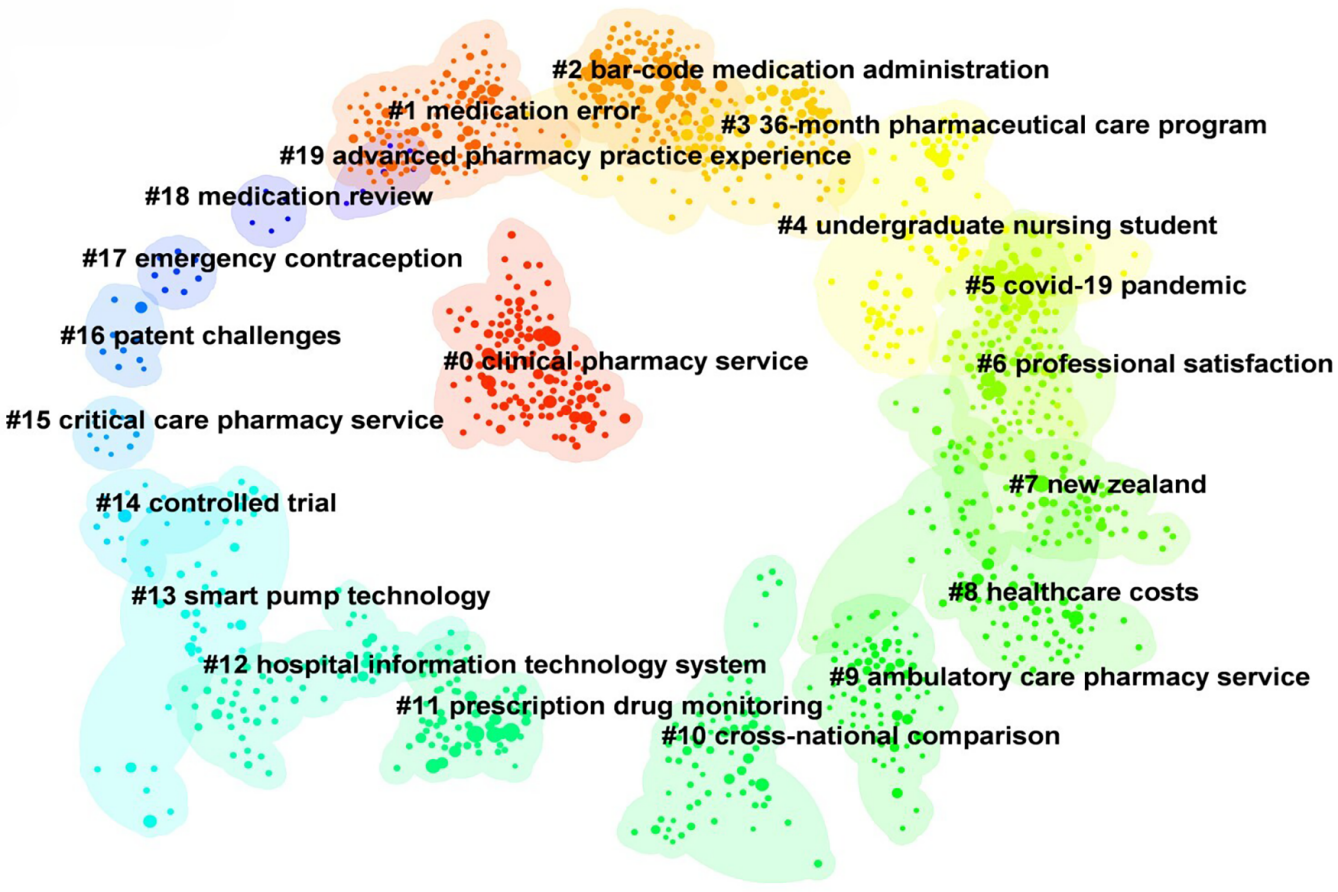
The literature co-citation network in the field of pharmacy management. Each color represents a different cluster. The top 20 cluster themes are: “clinical pharmacy service”, “medication error”, “bar-code medication administration”, “36-month pharmaceutical care program”, “undergraduate nursing student”, “COVID-19 pandemic”, “professional satisfaction”, “New Zealand”, “healthcare costs”, “ambulatory care pharmacy service”, “cross-national comparison”, “prescription drug monitoring”, “hospital information technology system”, “smart pump technology”, “controlled trial”, “critical care pharmacy service”, “patent challenges”, “emergency contraception”, “medication review” and “advanced pharmacy practice experience”.

Based on CiteSpace6.2.R4 (Advanced), the literature co-citation network was obtained (Figure 8), and the top 20 clusters were: “clinical pharmacy service” (*N* = 121, *S* = 0.901), “medication error” (*N* = 121, *S* = 0.958), “bar-code medication administration” (*N* = 113, *S* = 0.905), “36-month pharmaceutical care program” (*N* = 108, *S* = 0.909), “undergraduate nursing student” (*N* = 80, *S* = 0.913), “covid-19 pandemic” (*N* = 77, *S* = 0.989), “professional satisfaction” (*N* = 77, *S* = 0.949), “New Zealand” (*N* = 75, *S* = 0.968), “healthcare costs” (*N* = 75, *S* = 0.925), “ambulatory care pharmacy service” (*N* = 72, *S* = 0.987), “cross-national comparison” (*N* = 67, *S* = 0.994), “prescription drug monitoring” (*N* = 65, *S* = 0.946), “hospital information technology system” (*N* = 61, *S* = 0.990), “smart pump technology” (*N* = 38, *S* = 0.975), “controlled trial” (*N* = 17, *S* = 1), “critical care pharmacy service” (*N* = 10, *S* = 0.997), “patent challenges” (*N* = 9, *S* = 0.988), “emergency contraception” (*N* = 8, *S* = 1), “medication review” (*N* = 6, *S* = 1) and “advanced pharmacy practice experience” (*N* = 6, *S* = 1). The cluster labels in this paper were extracted by the Log likelihood ratio (LLR) algorithm. The N value is the capacity of each cluster, the silhouette (S value) is used to measure the homogeneity between nodes in each cluster, and an S value greater than 0.7 indicates that the clustering result is convincing.

By using the “cluster explore” function in CiteSpace, we obtained the citation information of the cocited networks (Table 2). In the “clinical pharmacy service” cluster, the strongly associated citation analyzed the clinical pharmacy service antitumor therapy interventions in a public tertiary hospital in Brazil through a retrospective study and evaluated the clinical significance and economic impact of clinical pharmacy services (30). In the “medication error” cluster, the strongly associated citation was a study on the incidence and preventability of adverse drug events in outpatient elderly patients (31). In the “bar-code medication administration” cluster, the strongly associated citation introduces the important role of barcode verification technology in improving medication safety (32). In the “36-month pharmaceutical care program” cluster, the strongly associated citation explored the effects of a 36-month medication care program on medication adherence in older patients with diabetes and hypertension (33).

**Table 2:** Literature co-citation network clustering information.

Cluster Id	Size	Silhouette	Top terms (LLR)	The strongly associated citation
#0	121	0.901	Clinical pharmacy service	da Rocha et al. (30)
#1	121	0.958	Medication error	Gurwitz et al. (31)
#2	113	0.905	Bar-code medication administration	Poon et al. (32)
#3	108	0.909	36-month pharmaceutical care program	Obreli-Neto et al. (33)
#4	80	0.913	Undergraduate nursing student	Schroers et al. (34)
#5	77	0.989	COVID-19 pandemic	Segal et al. (35)
#6	77	0.949	Professional satisfaction	Stuurman-Bieze Aet al. (36)
#7	75	0.968	New Zealand	Main et al. (37)
#8	75	0.925	Healthcare costs	Upadhyay et al. (38)
#9	72	0.987	Ambulatory care pharmacy service	Sturgess et al. (39)
#10	67	0.994	Cross-national comparison	Lancaster et al. (40)
#11	65	0.946	Prescription drug monitoring	Delcher et al. (41)
#12	61	0.99	Hospital information technology system	Li et al. (42)
#13	38	0.975	Smart pump technology	Giuliano et al. (43)
#14	17	1	Controlled trial	Murray et al. (44)
#15	10	0.997	Critical care pharmacy service	Rudis et al. (45)
#16	9	0.988	Patent challenges	Madden (46)
#17	8	1	Emergency contraception	Brandao (47)
#18	6	1	Medication review	Gurwitz et al. (48)
#19	6	1	Advanced pharmacy practice experience	Kassam et al. (49)

In the “undergraduate nursing student” cluster, the strongly associated citation is a descriptive statistical study on clinical nursing students at a private university in the Midwest of the United States, aiming to understand undergraduate nursing students’ experience in skill application in clinical practice, supervision quality and feedback (34). In the “COVID-19 pandemic” cluster, the strongly associated citation explored the experience and limitations of clinical pharmacists in Washington State in conducting telemedicine for chronic disease and cancer patients in the context of the COVID-19 pandemic (35). In the “professional satisfaction” cluster, the strongly associated citation is a study on clinical drug care interventions, which describes the drug care process tailored to the individual problems of patients in the randomized controlled trial intervention group and discusses the satisfaction of pharmacists with pharmaceutical care methods (36). In the “New Zealand” cluster, the strongly associated citation explores the policy mechanisms behind New Zealand's remarkable achievement in cost control of public pharmaceutical expenditure, which contrasts with most other advanced economies (37).

In the “healthcare costs” cluster, the strongly associated citation reported the positive impact of a pharmacist's supervised intervention on the direct medical cost burden of patients with newly diagnosed diabetes through a nonclinical randomized controlled trial approach (38). In the “ambulatory care pharmacy service” cluster, the strong association citation introduced the positive role of pharmaceutical care in community pharmacies in improving the medication compliance of elderly patients (39). In the “cross-national comparison” cluster, the strongly associated citation was a cross-country comparison between the United Kingdom and Australia in terms of “drug use problems” (40). In the “prescription drug monitoring” cluster, the strongly associated citation introduced the significant impact of Florida's prescription drug monitoring program (PDMP) in reducing oxycodone deaths in the state (41).

In the “hospital information technology system” cluster, the strongly associated citation introduced the application of computer information technology in hospital clinical nursing work. Based on the Internet of Things (IoT), this study optimized the infusion process through a reasonable and effective outpatient and emergency infusion drug management system, realized closed-loop drug management, and greatly improved the efficiency and safety of infusion (42). In the “smart pump technology” cluster, the strongly associated citation introduced the positive role of intravenous (IV) smart pumps in reducing dose errors and drug administration error rates, identifying system errors, and identifying nurse training needs. These findings show the broad application prospects of medical information technology in the field of improving clinical patient satisfaction and drug safety (43). In the “controlled trial” cluster, the strongly associated citation was a randomized trial study of pharmacist intervention to improve medication adherence in patients with heart failure (44). In the “critical care pharmacy service” cluster, the strongly associated citation was a position paper on critical care pharmacy services, and the article identified and described the scope of practice of acute critical pharmacy pharmacists and acute critical pharmacy services (45).

In the “patent challenges” cluster, the strongly associated citation introduced the interaction between intellectual property rights and the pharmaceutical regulatory system. The article identifies problems with the current pharmaceutical regulatory system operating independently of the intellectual property system and describes the attempts and experiences of many regulators in developed countries to ensure a compromise between the rights of generic companies and intellectual property owners by incorporating safeguards, such as patent linkage and data protection, into the regulatory framework (46). In the “emergency contraception” cluster, the strongly associated citation was a social survey study on the issue of pharmacists’ and sales clerks’ positions on emergency contraception, which discussed pharmacists’ views on the commercialization of emergency contraception in Brazil and on the issue of pharmaceutical care for emergency contraceptive users (47). In the “medication review” cluster, the strongly associated citation was a study on adverse drug events, which focused on the incidence and prevention of adverse drug events in outpatient care for the elderly (48). In the cluster of “advanced pharmacy practice experience”, the strongly associated citation is a study on the cultivation of pharmaceutical care ability. This paper focuses on the nature and degree of learning opportunities that pharmacy students have in advance in the community (49).

**Figure 9:**
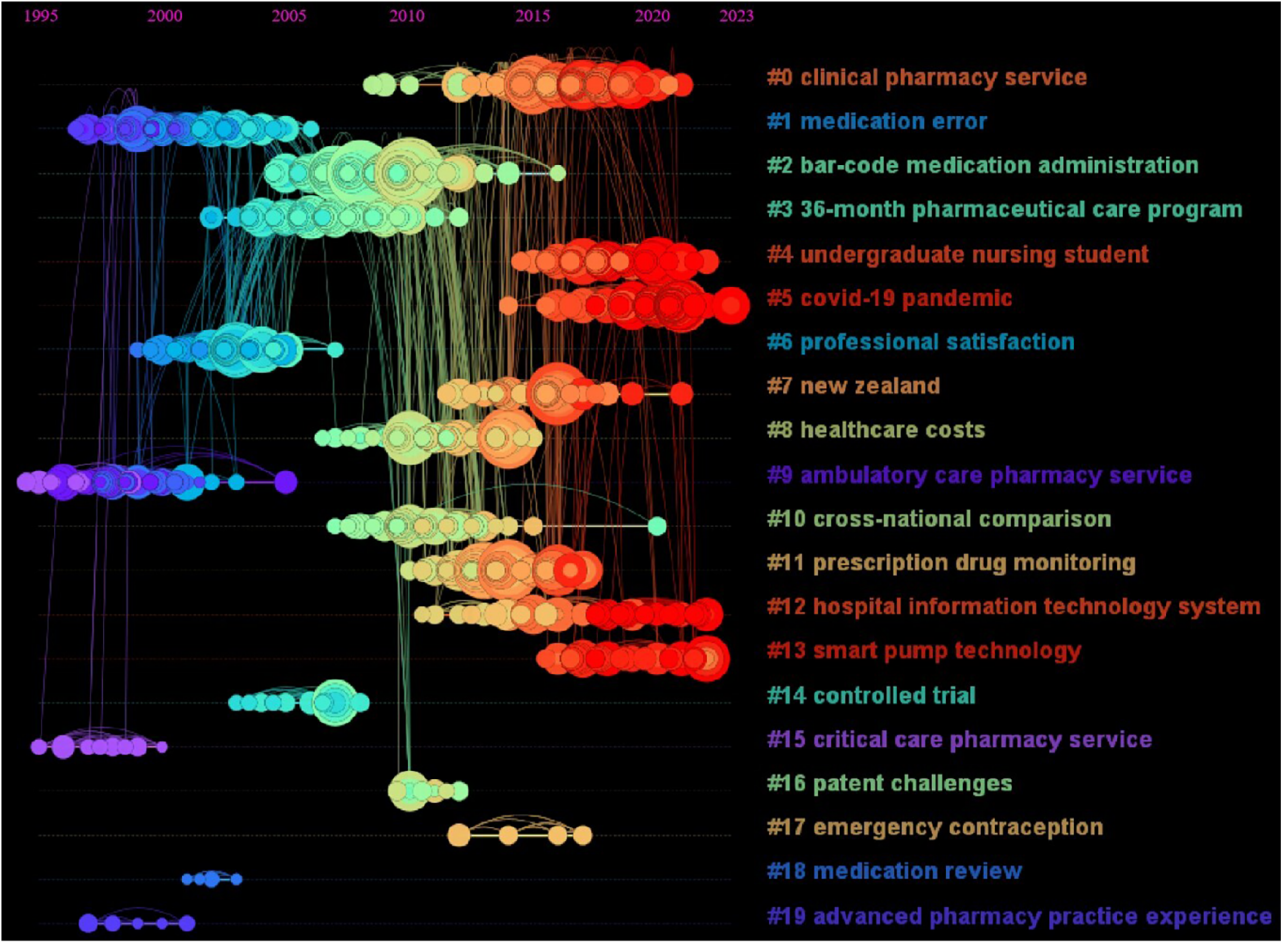
Time zone map of the literature co-citation network. The time zone map reflects the distribution of all cited literature nodes in each cluster in the time dimension, and then maps the evolution trend of hot topics in the field.

Based on the literature co-citation network, we constructed a citation distribution map of the time series (Figure 9). According to the results, these citations from 1995 to 2005 were mainly distributed in the “medication error”, “professional satisfaction”, “ambulatory care pharmacy service”, “critical care pharmacy service” and “advanced pharmacy practice experience” clusters, which were the hot research directions in this period. From 2005 to 2015, these citations were distributed mainly among “barcode medication administration”, “36-month pharmaceutical care program”, “healthcare costs”, “cross-national comparison”, “prescription drug monitoring”, “patent challenges” and “emergency contraception”. From 2015 to 2023, these citations were distributed mainly in the “clinical pharmacy service”, “undergraduate nursing student”, “COVID-19 pandemic”, “New Zealand”, “hospital information technology system” and “smart pump technology” clusters and are hot topics.

### 3.5 Burst detection

Burst detection is mainly used to mine keywords or cited literature with large frequency fluctuations over a period of time. Through burst detection, we can discover the evolutionary trend of the field of pharmaceutical management from macro to micro, from single to multiple, and review or predict which key technology branches will become hot topics of concern in the future. Based on CiteSpace 6.2.R4, we conducted a burst detection of keywords in these literatures. After detection, a total of 217 keywords were found to have citation bursts. To ensure the reliability of the detection results, this article uses the keyword mutation time as the basis for sorting, and the 25 nodes with the largest mutation intensity are selected.

**Figure 10:**
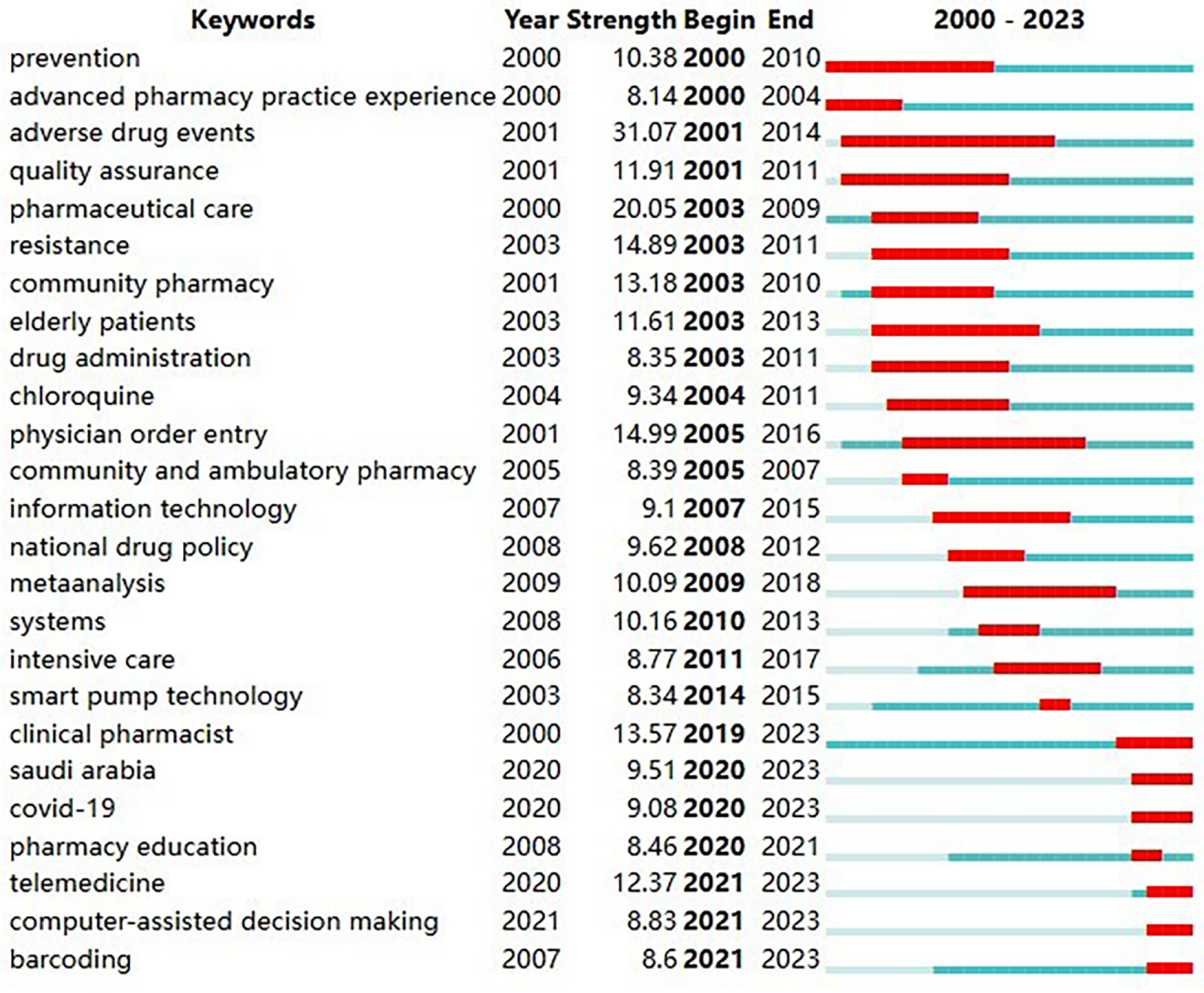
Top 25 keywords with the strongest citation bursts. The keywords “clinical pharmacist”, “Saudi arabia”, “COVID-19”, “pharmacy education”, “telemedicine”, “computer-assisted decision making” and “barcoding” have shown high burst intensity in recent years. “Begin” represents the start time of the keyword burst, “End” represents the year when the burst ended, and “Strength” represents the keyword burst intensity. The larger the value is, the larger the frequency fluctuation. The red bands represent the length of the burst.

The results are shown in Figure 10, in which “Begin” represents the start time of the keyword burst, “End” represents the year when the burst ended, and “Strength” represents the keyword burst intensity. The larger the value is, the larger the frequency fluctuation. The red bands represent the length of the burst. From the perspective of time series, the keywords “prevention”, “advanced pharmacy practice experience”, “adverse drug events”, “quality assurance”, “pharmaceutical care”, “resistance”, “community pharmacy”, “elderly patients”, “drug administration”, “chloroquine” and “community and ambulatory pharmacy” had higher burst intensities in the 2000–2010 phase. Keywords such as “information technology”, “national drug policy”, “meta analysis”, “systems”, “intensive care” and “smart pump technology” had high burst intensities during the period 2011–2019. Keywords such as “clinical pharmacist”, “saudi arabia”, “COVID-19”, “pharmacy education”, “telemedicine”, “computer-assisted decision making” and “barcoding” had high burst intensities from 2020 to 2023. It should be noted that the keyword “saudi arabia” here refers to the related pharmaceutical management policy (50), pharmaceutical care (51), pharmaceutical education (52) and other related research topics with a significant increase in frequency during this period.

**Figure 11:**
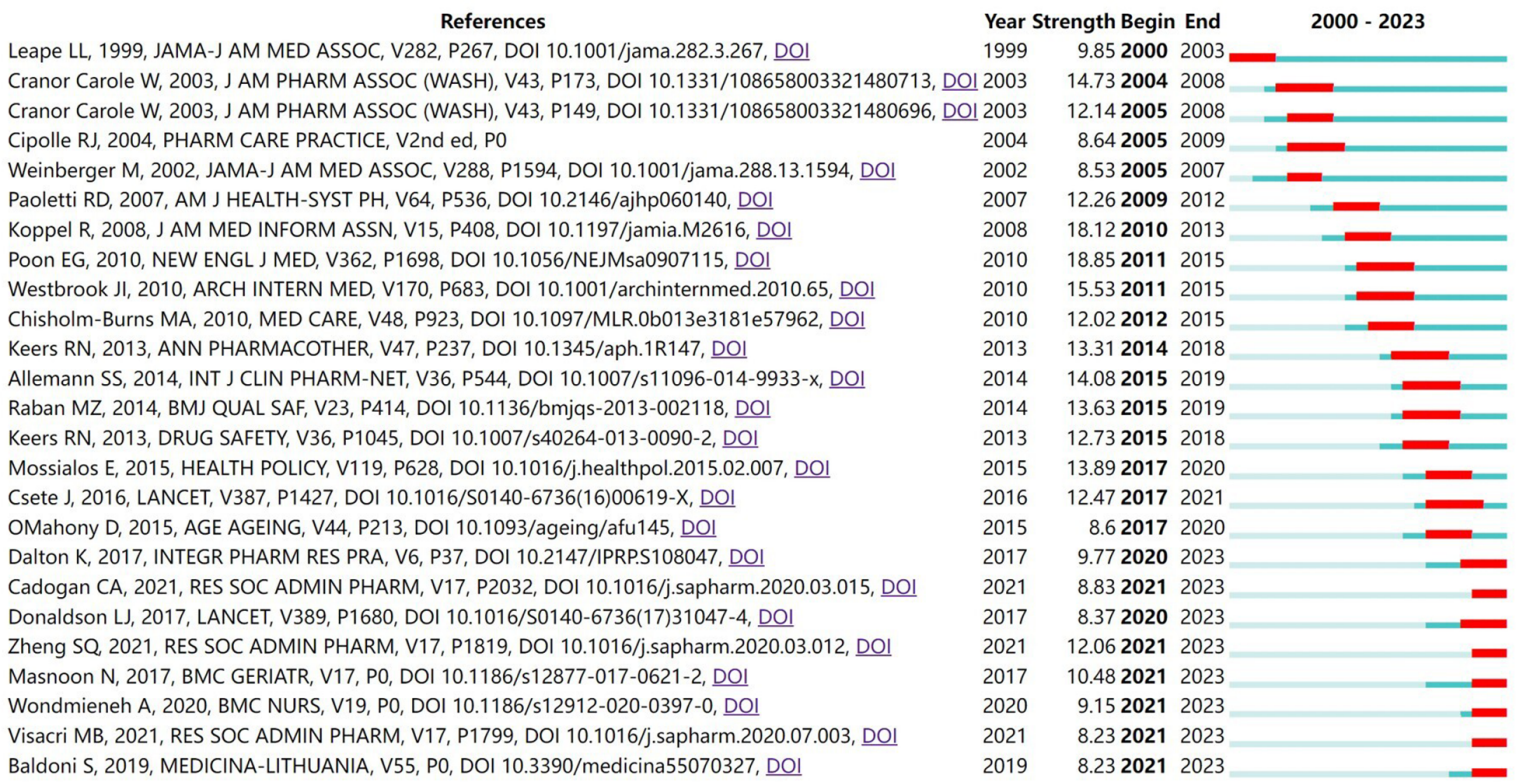
Top 25 references with the strongest citation bursts. “Begin” represents the start time of the reference burst, “End” represents the year when the burst ended, and “Strength” represents the reference burst intensity. The larger the value is, the larger the frequency fluctuation. The red bands represent the length of the burst.

Based on the “Burstness” function, a total of 247 references had citation bursts, and the 25 nodes with the largest burst intensity are displayed (Figure 11). From 2000 to 2009, research content with a high burst of citations focused on the positive impact of pharmacist reviews on reducing medication errors (53) and the contribution of community pharmacists to improving clinical outcomes and reducing healthcare costs (54, 55). From 2010 to 2019, the research content with a high burst of citations focused on the impact of electronic medication records (EMAR) and barcode medication administration (BCMA) technology on medication safety (32, 56, 57), the verification of the correlation between medication interruptions and the risk level of medication errors (58, 59), the positive impact of pharmacists on patient care (60) and chronic disease management (61), the definition of pharmaceutical services (19), and the prevalence, nature (62) and causes (63) of medication errors in health care settings. From 2020 to 2023, the research content with a high burst of citations focused on the role of pharmacists in reducing medical costs (64), the contribution of community pharmacists to the COVID-19 pandemic (28, 65, 66), the definition of polypharmacy (67), medication safety (68), medication error factors and improvement (69), and telepharmacy services (70).

## 4 Discussion

### 4.1 Answer for Q1: what are the trends in publications in the field of pharmaceutical management?

In the Web of Science Core Collection database, the publication of papers in the field of pharmacy management first started in 2000, with only 130, peaking at 1,335 in 2021 and then declining slightly, with a total of 12,771 publications accumulated during this period. According to the results of keyword co-occurrence analysis and literature co-citation analysis, the early studies mainly focused on the positioning of pharmacists and pharmaceutical care and the establishment of management system, which also provided a rich theoretical basis for subsequent practical research in the field of pharmaceutical management. With the intensification of global health challenges, the extensive application of information technology in the medical field and the continuous changes in the policy environment, pharmaceutical management research has ushered in a rich and diverse practical scenarios and theoretical exploration space, which has contributed to the significant growth of the literature in the field. Notably, these literatures were widely distributed in the two subject clusters of biology and medicine, psychology and social sciences, and the core involved pharmacology & pharmacy, health care science services, public environmental occupational health, substance abuse, nursing, health policy services, education science disciplines, and medical informatics.

To further evaluate the relativity of this growth phenomenon, we compared the publication trend of literature in fields close to pharmaceutical administration, including public health emergency management (71), global health management (72), pharmaceutical services (7), and pharmaceutical economics and policy research (6). The results showed that although the growth rate of literature publication in each field varied, it generally showed an upward trend similar to that in the field of pharmacy management. This shows that the growth of literature in the field of pharmaceutical administration is not an isolated phenomenon, but coincides with the booming background of the entire medical and health research field, which also reflects the general increase in attention to the related fields of drug regulation worldwide.

### 4.2 Answer for Q2: what are the most prolific and influential countries/regions and institutions in this field?

Generally, the annual publication volume and citation frequency of scholars or scientific research institutions are the easiest to quantify, and they are also the most intuitive indicators reflecting their academic influence (73). According to the map of national cooperation network in Figure 4, the United States ranks first in the world in terms of the number of publications (*n* = 4,557) and citations (*n* = 106,685), and radiates to Britain, Canada, Australia, China, Spain, Brazil, the Netherlands, Germany, France and Italy in terms of cooperation. Reflecting its significant academic influence in the field of pharmaceutical administration, it also means that the United States plays a key role in setting international drug standards, promoting transnational drug safety regulatory information exchange, and improving global access and quality of drugs, which plays a crucial role in the standardization and efficiency of global pharmaceutical practice. In addition, in terms of institutional cooperation network (Figure 5), the University of Toronto ranked first among all institutions in terms of the number of publications (*n* = 242) and citations (*c* = 8,973), and radiated to other institutions.

This cooperation pattern is not only a reflection of academic influence, but also a vindicator of the dynamic evolution of the global drug regulatory system. These data underscore the importance of stronger international cooperation, particularly to facilitate rapid response mechanisms and accelerate the development and distribution of vaccines and therapeutics in the face of global public health challenges, such as COVID-19. In the future, the research in the field of pharmaceutical administration should pay more attention to interdisciplinary and cross-border cooperation, and promote the continuous improvement of the global drug regulatory system based on scientific evidence.

In this study, the analysis of cooperation in the field of pharmacy management mainly focused on the description and analysis of the number of publications jointly published by countries or institutions. A notable limitation is that we failed to take into account the distribution of the number of HEIs within countries or the effect of the size of individual institutions on the output of research outcomes. For example, although the United States leads in total publications and the University of Toronto stands out among institutions, the international collaborative impact rankings might look different if we could obtain and analyze data on the number of universities or colleges in each country and the size of their corresponding research departments. Because of practical difficulties in obtaining such detailed data, we were not able to include such adjustment factors directly in our analyses, which constitutes a limitation of the study. Future research could explore how these variables can further refine and influence the analysis of the international cooperation landscape through more extensive data integration.

### 4.3 Answer for Q3: what are the main research directions and hotspots? how did they change over time?

Through keyword co-occurrence analysis (Figure 6) and literature co-citation analysis (Figure 8), we explored the distribution of the core research topics of papers in the field of pharmaceutical management from the perspectives of keywords and cited literature. The findings can be summarized as follows: pharmacy services and clinical pharmacists, development and implementation of drug policies and regulations, drug utilization evaluation, medical insurance and drug cost control, medication safety and risk management, drug information management and technology application, pharmacoeconomics and drug evaluation, education and training of pharmacy students, career satisfaction of pharmacists, pharmaceutical intellectual property protection, and drug review.

As shown in the co-cited network time zone map (Figure 9), early pharmacy management research (1995–2000) focused on the positioning of pharmacists and pharmacy services and the establishment of management systems. The research content covered the important role of pharmaceutical services in clinical practice, the positioning of pharmacists and pharmaceutical education. From 2000 to 2005, research was conducted mainly in clinical pharmacy and institutional regulation, with research topics covering pharmacists’ professional satisfaction, clinical medication guidance and clinical trials. With the development of globalization and the market economy, pharmaceutical administration research began to shift to the field of drug marketing and economics from 2005 to 2010. The research topics included drug market competition, drug intellectual property protection, drug consumption behavior, drug cost‒benefit analysis, medical insurance, etc. In 2010–2015, pharmaceutical management research was gradually integrated into the field of health systems and healthcare delivery, with research topics including healthcare quality and safety (e.g., medication safety), healthcare service delivery, and patient rights protection. With the rapid development of digital technology, research in the field of pharmacy management has also begun to explore the direction of digitalization and intelligence since 2015, and the research topics include digital drug management, intelligent medical services, and telemedicine. In view of the COVID-19 global pandemic, the research hotspots in 2000–2023 have focused mainly on the research directions of drug supply management, pharmacy services and telemedicine services under major public health events.

### 4.4 Answer for Q4: what are the current research frontiers in the field? what are the potential hotspots for the future?

Through the burst detection of keywords (Figure 10) and co-cited literature (Figure 11), we found that the topics of telepharmacy services, information technology, computer-assisted decision making, pharmacy education, and cross-national comparisons of pharmacy policies showed a high burst intensity. Therefore, it is predicted that future research in the field may focus on the following areas.

I. Information technology and pharmacy: With the development of information technology, the application of big data, artificial intelligence and other technologies in the medical field will further increase. Pharmacy management will focus more on utilizing these technologies to improve the efficiency and quality of medical management. For example, big data are used for the monitoring, analysis and management of adverse drug reactions (74–76), and artificial intelligence is used for prescription reviews and medication reminders (77, 78).
II. Precision pharmacy: Precision Pharmacy is an emerging field of multidisciplinary crossover between pharmacy management and clinical pharmacy, bioinformatics and other disciplines. It aims to provide patients with personalized and precise drug use solutions based on their genome, phenotype and other information. In the future, more in-depth research on precision pharmacies, including research on pharmacogenomics, precision drug use decision-making, and precision clinical trials, will be performed (79).
III. Global Pharmaceutical Governance: With the deepening of globalization and international exchanges, global pharmaceutical governance has become an important topic in pharmaceutical management. This includes comparative research on international drug regulation, transnational drug circulation and trade rules (80, 81). In addition, with the deepening of the Belt and Road Initiative, the study of drug regulatory systems and policies of countries along the route will also become a priority.
IV. Pharmaceutical ethics and legal issues: With the development of pharmaceutical science and technology, pharmaceutical ethical and legal issues have become increasingly prominent. In the future, ethical and legal research on these issues, such as gene editing in the field of pharmaceutical research and artificial intelligence-assisted decision-making, will become popular (82, 83). In addition, research on the protection of pharmaceutical intellectual property rights (84) and data privacy (85) will also become a focus of attention.

## 5 Conclusion

Based on the macro exploration of bibliometric analysis, this study revealed the research pattern and development trend in the field of pharmacy management, highlighting the importance of this field in addressing complex and changing health challenges. The trend of diversification of research topics, It covers the optimization of clinical pharmacists and pharmaceutical services, efficient evaluation of drug utilization, fine control of medical insurance and drug costs, strengthening of drug safety and risk management system, innovation and application of drug information management technology, deepening of pharmacoeconomic evaluation, expansion of drug intellectual property protection strategies, and continuous improvement of pharmaceutical education and regulatory framework. These constitute the core pillars of pharmaceutical administration research.

Current research focuses on the modernization transformation of pharmaceutical education, especially in the training of future pharmaceutical professionals. The comprehensive drug management strategy in the context of COVID-19 highlights the key role of pharmaceutical management in public health emergency response; The development and application of digital drug management system is leading the practice of pharmacy management into a new era of intelligence; And the expansion of telemedicine services has opened up a new way to improve the accessibility of medical services for patients. These hot topics not only reflect the characteristics of pharmaceutical management research keeping pace with The Times, but also provide scientific basis and guidance for the current pharmaceutical practice.

Looking forward to the future, research front pointing in the direction of information technology and drug management innovation mode of depth fusion, such as precision of pharmaceutical care, heralds the arrival of the era of personalized medicine; Global drug management system construction and cooperation, to ensure global drug supply security, promote health equity have far-reaching significance; Under the background of the rapid development of artificial intelligence technology, the ethical and legal considerations of drug design have become a new issue that needs to be solved urgently, which requires the interdisciplinary integration of pharmaceutical administration research to meet the challenges of science and technology ethics.

## 6 Limitations

Due to the limitations of the overall structure and length of the article, this study only provides a macro overview of the field of pharmacy management, without deeper discussion of specific subtopics. We encourage scholars to select the subfields of interest for subsequent thematic research according to the outline outlined in this study, and we also plan this as part of future personal research plans.
